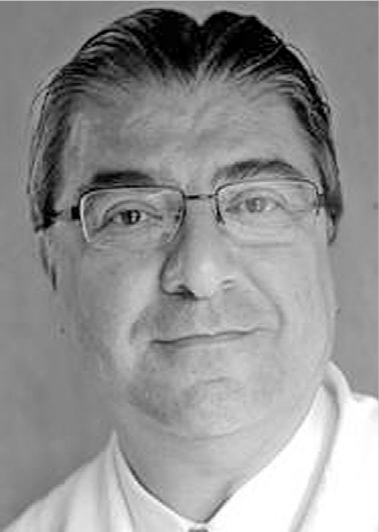# Biocatalytic and Bioelectrocatalytic Approaches for the Reduction of Carbon Dioxide using Enzymes

**DOI:** 10.1002/ente.201600610

**Published:** 2017-01-20

**Authors:** Stefanie Schlager, Angela Dibenedetto, Michele Aresta, Dogukan H. Apaydin, Liviu M. Dumitru, Helmut Neugebauer, Niyazi S. Sariciftci

**Affiliations:** ^1^ Linz Institute of Organic Solar Cells (LIOS) Johannes Kepler University Linz Altenbergerstraße 69 4040 Linz Austria; ^2^ Department of Chemistry and CIRCC University of Bari, Campus Universitario via Orabona 4 70126 Bari Italy; ^3^ Chemical Engineering Faculty University of St. Bath Bath UK

**Keywords:** biocatalysis, dehydrogenases, electroenzymatic processes, enzymes, reduction

## Abstract

In the recent decade, CO_2_ has increasingly been regarded not only as a greenhouse gas but even more as a chemical feedstock for carbon‐based materials. Different strategies have evolved to realize CO_2_ utilization and conversion into fuels and chemicals. In particular, biological approaches have drawn attention, as natural CO_2_ conversion serves as a model for many processes. Microorganisms and enzymes have been studied extensively for redox reactions involving CO_2_. In this review, we focus on monitoring nonliving biocatalyzed reactions for the reduction of CO_2_ by using enzymes. We depict the opportunities but also challenges associated with utilizing such biocatalysts. Besides the application of enzymes with co‐factors, resembling natural processes, and co‐factor recovery, we also discuss implementation into photochemical and electrochemical techniques.

## 1 Introduction

Already in 1896, Svante Arrhenius discussed in his work the impact of atmospheric carbon dioxide (CO_2_) on the greenhouse effect. According to his calculations, Arrhenius stated even back then a correlation between the CO_2_ content in the atmosphere and an increase in the Earth's temperature.^1^ Nowadays, concerns regarding greenhouse gases, particularly CO_2_, and global warming affect politics, economy, and society. In comparison to other greenhouse gases such as methane (CH_4_) and water vapor, CO_2_ has the highest impact on global warming, as its atmospheric residence time is the highest, and moreover, its content in the atmosphere is second only to water vapor.^2^, ^3^

CO_2_ is generated from the combustion of fossil carbon (e.g., oil, gas, coal) and biomass in which energy is released. Owing to the finite reserve of fossil‐C, another issue is now rising: the convenience to recycle carbon more than to release it into the atmosphere or dispose of it underground. On the basis of these facts, primarily the utilization of CO_2_ and substitution of fossil fuels as energy carriers have become some of the most discussed topics and have especially drawn attention from the scientific community.^4^, ^5^

## 2 CO_2_ as Chemical Feedstock

To reduce atmospheric CO_2_, two approaches comprising different techniques are generally considered. In the carbon capture and sequestration (CCS) approach, CO_2_ is stored in deep rock cavities under sea and land.^6^ Practice of course is not ubiquitous nor accepted by the public everywhere and does not utilize CO_2_ as such. Differently, in the carbon capture and utilization (CCU) approach, CO_2_ is regarded as a carbon feedstock and starting material for artificial fuels and chemicals. With this strategy, both issues, that is, depletion of fossil fuels and reduction of CO_2_ in the atmosphere, are taken into account. In this work, we will therefore address CCU as a key target to substitute fossil fuels and to reduce the atmospheric CO_2_ content at the same time.^7^, ^8^, ^9^, ^10^, ^11^, ^12^

**Figure 1:**
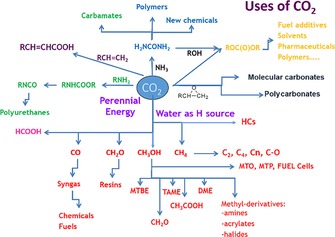
Possible reaction routes and materials from CO_2_ as feedstock in the CCU approach.

From a chemical point of view, CO_2_ is a highly stable molecule in which the carbon atom is in a +4 oxidation state (Figure 1).^13^ Any conversion of CO_2_ into a species in which the carbon atom maintains the +4 oxidation state is an exergonic process (right part of Figure 1); conversely, any conversion into a molecule in which the carbon atom has a lower oxidation state (+2 or even lower, left part of Figure 1) requires energy. Fuels fall into this latter category (lower part of Figure 1). Noteworthy, to produce energy‐rich chemicals, energy and hydrogen are necessary.^14^ The latter must be generated from water by using perennial energies such as sun, wind, hydropower, and geothermal (SWHG) energies. This is a must. Therefore, perennial SWHG energies must be used to convert large volumes of CO_2_ into energy‐rich products such as fuels.

Table 1 depicts possible reduction reactions of CO_2_ into various possible products through one, two, or more electron‐transfer reactions. For each reaction, standard thermodynamic reduction potentials are reported for aqueous solutions at pH 7. They give an idea of the required energy input for the conversion of CO_2_ into a certain product.

**Table 1:** Theoretical formal reduction potentials (*E* ^0^′) of CO_2_ to various products through two or multielectron reduction reactions. The potentials are calculated for aqueous solutions at pH 7.

Reaction					*E* ^0^′ [V]
CO_2_		+1 e^−^	→	CO_2_^.−^	−1.90
2 CO_2_		+2 e^−^	→	CO+CO_3_^2−^	−1.33
CO_2_	+2 H^+^	+2 e^−^	→	CO+H_2_O	−0.53
CO_2_	+2 H^+^	+2 e^−^	→	HCOOH	−0.61
CO_2_	+4 H^+^	+4 e^−^	→	H_2_CO+H_2_O	−0.48
CO_2_	+6 H^+^	+6 e^−^	→	CH_3_OH+H_2_O	−0.38
CO_2_	+8 H^+^	+8 e^−^	→	CH_4_+2 H_2_O	−0.24

In any practical approach, however, the above potentials or energy inputs are expected to be higher. In fact, to perform CO_2_ conversion, overpotentials have to be considered. To lower those energy barriers, reaction conditions such as high potentials, high pressures, and/or low temperatures^15^, ^16^ are required or catalysts have to be utilized to perform the reduction at a potential as close as possible to the thermodynamic value.^17^

However, whereas the incorporation of CO_2_ into cyclic carbonates^18^ (for application in cosmetics or adhesives) or polymers,^19^ in which CO_2_ is an essential feedstock for industrial applications, is a low‐energy process, the production of artificial fuels from CO_2_ is a process that requires a high energy input, even if it has great interest for the larger volume of fuels with respect to chemicals and materials.^20^, ^21^, ^22^

**Figure 2:**
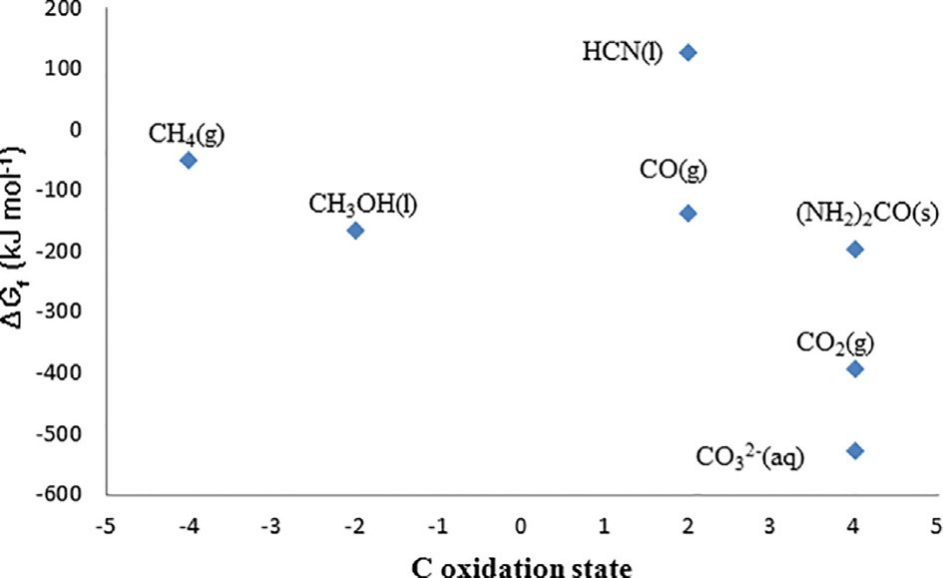
Gibbs free energies of C_1_ molecules.^13^

Considering the possible products that can be generated from the reduction of CO_2_, alcohols specifically emerge as eminently advantageous. In particular, the C_1_ and C_2_ products methanol and ethanol are highly desired, as they meet the requirement of direct application as fuels. However, even though higher alcohols would provide higher energy densities for fuel applications, the obtainment of mainly C_1_ compounds such as methanol is thermodynamically favored from the chemical reduction of CO_2_ (see Figure 2 and Table 1).^23^ The challenge for this is to find techniques and/or catalysts to lower the energy barrier for the reduction reaction and to enable operation under rather mild reaction conditions.

To reduce CO_2_ chemically, electron sources or donors (sacrificial) are required. Besides photochemical and photoelectrochemical approaches, which mimic artificial photosynthesis,^24^, ^25^ electrochemical techniques^26^, ^27^ have also aroused interest. All of these strategies, however, involve electron injection from an energy source or result from excitation from a light source. Either way, the energy must be provided from perennial energy sources as discussed above, indirectly by driving electrochemical processes or directly by irradiation.^28^

For this purpose, bioinspired materials, based on models from photosynthesis and other biological approaches, and biobased materials have particularly gained high interest. Besides several approaches involving the use of organic and metal–organic compounds as catalysts in photochemical, photoelectrochemical, and electrochemical methods, the direct application of biocatalysts such as enzymes and microorganisms is, above all, favored for utilization in CO_2_ reduction.^29^, ^30^, ^31^ The main advantages of biocatalysts, in comparison to synthetic catalysts, are high selectivity towards the products obtained and high yields.^32^ Furthermore, as natural processes mainly proceed under ambient conditions, the utilization of biocatalysts eases processes in terms of conditions and makes them highly attractive for possible large‐scale applications.^33^ Enzymes especially feature remarkable potential for this purpose, as they are nonliving and, therefore, do not require nutrients or have to be especially treated in contrast to microorganisms including algae. Also, processes involving the use of living organisms underlie self‐regeneration or replication, which is additionally dependent on environmental conditions such as nutrients, temperature, and pH value. Moreover, microbial CO_2_ conversion is mainly based on fermentation processes, and as such, those factors pose problems for scaling for biofuel production. Isolation of individual enzymes from living organisms would, therefore, be an attractive alternative.^34^, ^35^

However, the properties of the enzyme as well as the desired reaction depend on the source of the enzyme or the microorganism from which it was isolated, and therefore, the microbial source has to be chosen accurately. For the direct reduction of CO_2_, dehydrogenases have particularly gained high interest. Moreover, dehydrogenase enzymes are capable of converting CO_2_ into alcohols directly under ambient conditions and in aqueous environments.^36^, ^37^, ^38^, ^39^

In the following paragraphs, enzymatic processes for CO_2_ reduction to different products and through various pathways will be discussed.

## 3 Enzymatic Catalysis for CO_2_ Conversion

### 3.1 CO_2_ reduction with co‐enzymes

As this review focuses on reduction of CO_2_, emphasis will be put on dehydrogenase enzymes.

Dehydrogenase‐catalyzed reactions can be performed either for reduction or for oxidation processes. Natural oxidation reactions preferably occur.^40^, ^41^ However, reaction kinetics can be influenced and reaction equilibria can be shifted by providing the substrate to be converted in excess amount and further by adding the corresponding redox equivalent of the co‐factor, which is required for charge and proton transfer.^42^ In this study, we mainly focus on the reductive pathway of enzymatic reactions to convert CO_2_.

**Scheme 1:**
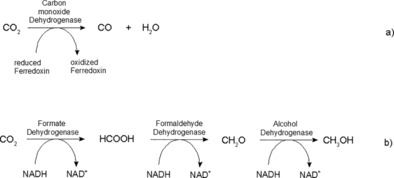
Reaction steps for the enzyme‐catalyzed reduction of a) CO_2_ to CO with carbon monoxide dehydrogenase and b) CO_2_ to CH_3_OH through a three‐step cascade of dehydrogenases.

For the direct reduction of CO_2_ there are two main possibilities involving the use of dehydrogenases, as shown in Scheme 1: first, CO_2_ can be reduced to carbon monoxide (CO) by utilizing carbon monoxide dehydrogenase (Scheme 1 a).^37^, ^43^, ^44^ Furthermore, conversion of CO_2_ into methanol is feasible by using a three‐step enzyme cascade including formate dehydrogenase (FDH), formaldehyde dehydrogenase (F_ald_DH), and alcohol dehydrogenase (ADH) (Scheme 1 b).^36^, ^45^

Both kinds of reactions require a sacrificial co‐factor that, in the case of reductions, serves as the electron and proton donor and that is, therefore, oxidized in the same step. Redox reactions involving the use of enzymes as catalysts occur through the formation of an intermediate compound, consisting of the enzyme, co‐factor, and substrate to be reduced or oxidized. Within this intermediate state, charge and proton transfer is performed between the co‐factor and substrate over the (metal) active site of the enzyme.^46^ The reduced substrate and oxidized co‐factor, or vice versa, are then released again.

In the case of most reactions involving the use of carbon monoxide dehydrogenase, ferredoxin serves as the electron and proton donor. Differently for formate, formaldehyde, and alcohol dehydrogenases, nicotinamide adenine dinucleotide (NADH) is the corresponding co‐enzyme. Each of the three steps in the cascade represents a two‐electron reduction and, therefore, needs one NADH molecule for each step to donate electrons and protons. Three molecules of NADH are therefore oxidized in the reduction of CO_2_ to CH_3_OH. In natural processes, the oxidized forms of the co‐factors are regenerated in subsequent redox reactions to complete a reversible cycle of reductions and oxidations. For approaches performed in vitro, however, those co‐factors are sacrificial and have to be delivered after the reaction or have to be regenerated in additional processes.

One of the first works involving the use of a dehydrogenase enzyme for the conversion of CO_2_ was done by Rusching et al. They report CO_2_ reduction to formate with NADH as the co‐factor. In their work, they perform homogeneous catalysis by dissolving the enzyme in solution and determine the formate generated by ^14^C labeling with ^14^CO_2_.^47^ In a different work, Schuchmann and co‐workers deal with CO_2_ hydrogenation by using a hydrogen‐dependent CO_2_ reductase (HDCR) isolated from the acetogenic bacterium *Acetobacterium woodii*. In this hydrogenation reduction to formate, formate dehydrogenase plays a key role.^48^ Considering the importance of the microbial source of the enzyme, Alissandratos has presented results on improved catalytic properties for CO_2_ reduction by using a formate dehydrogenase expressed from the *Clostridium carboxidivorans* strain P7^T^.^49^ However, an important point for the efficient and sustainable use of enzymes in experimental approaches is the fact of denaturation of enzymes, due to their delicate nature, if used as homogenous catalysts in solution.

One possibility to improve thermal stability is the application of thermophilic enzymes, as discussed by Honda et al.^50^ Moreover, apart from thermal stabilization, it has been found that suitable immobilization of enzymes prevents their degradation and enables further reusability of the catalyst and their easier separation from products.^51^ However, a crucial thing here is to find appropriate materials that do not limit mass transport of substrate to the catalyst active site and release of the product from the system. Heichal‐Segal and co‐workers first investigated an alginate–silicate hybrid matrix for the immobilization of glucosidase. They report promising results that show that the activity is not leached and chemical and thermal denaturation of the enzymes can be avoided.^52^ This matrix has also turned out to be highly suitable for dehydrogenases. Obert and Dave show the application of alginate–silicate hybrid gel for the three‐step dehydrogenase cascade (se Scheme 1) to reduce CO_2_ to methanol.^36^ Moreover the groups of Xu and Lu have investigated such hybrid gels for the conversion of CO_2_ into formate by using formate dehydrogenase and have also extended the cascade to methanol generation. They describe an optimized constitution between alginate and silicate and reveal the importance of silicate for cross‐linking in the gel. As a silicate source, they use tetramethoxysilane [Si(OCH_3_)_4_].^45^, ^53^ This, however, may lead to methanol release if not sufficiently hydrolyzed in the liquid phase for the subsequent bead‐formation procedure. Differently, Aresta et al. use tetraethoxysilane [Si(OC_2_H_5_)_4_] instead to avoid such interferences.^54^

In a different study, Luo et al. show the sequential and co‐immobilization of all three dehydrogenases for the reduction of CO_2_ to methanol. In their work, they present immobilization by noncovalent binding to flat‐sheet polymeric membranes. They report not only promising results for both immobilization techniques but even better methanol yield from sequential immobilization.^55^

All these results emphasize that immobilization of enzymes offers the possibility for the efficient use of enzymes owing to enabled reusability, reproducibility, and improved stability.

Even though CO_2_ reduction reactions with dehydrogenases together with the corresponding co‐factors provide products in high yields and high selectivity, such reactions are limited to laboratory‐scale applications owing to the high costs of the co‐factor supply and regeneration.

For economically favorable use and subsequent potential large‐scale utilization, the framework conditions of the enzymes, in general, have to be considered. For this, the whole reaction mechanism, including parameters such as temperature, pressure, pH, solvent, and co‐factors, has to be taken into account. Specifically, the role of the co‐factors is crucial and has a particular impact on cost and efficiency of the enzymatic processes. As discussed earlier in this study, co‐enzymes and co‐factors are usually sacrificial for redox reactions and are therefore depleted. Nature has developed their recycling by using other enzymes that may regenerate the reduced forms.

Nevertheless, to avoid co‐factor loss in technical implementations and to improve the efficiency of these redox processes, several approaches have been developed. Some of them focus on co‐factor regeneration or substitution, which will be discussed in the following paragraphs.

### 3.2 Co‐enzyme regeneration

One strategy to make enzymatic CO_2_ reduction feasible and attractive for large‐scale application is co‐factor substitution or regeneration. Depending on the desired reaction, either the oxidized or the reduced form of the co‐factor is required. Therefore, in the case of regeneration strategies, additional redox processes are established, for which the co‐factor is reduced or oxidized to its initial state and then becomes reusable for the actual redox reaction. Aksu et al. have published results showing the regeneration of oxidized nicotinamide co‐factors. They compare results for NADH and its phosphorylated derivative NADPH. They couple an alcohol dehydrogenase catalyzed oxidation reaction to a laccase oxidation with simple O_2_ that, moreover, provides the electron for reduction and, therefore, allows regeneration of the co‐factor. For both derivatives, they find similar rates and turnover numbers over 300.^56^ Zhang et al. show an approach to substitute NAD(P)H co‐factors with an artificial fluoro‐containing co‐factor based on a simplified structure.^57^ Also, Paul and co‐workers report work on simplified synthetic co‐factors comprising structures of NAD(P)H.^58^ Work on co‐factor regeneration has been presented, for example, by Palmore and co‐workers. The topic of their study is the regeneration of the oxidized form NAD^+^.^59^

However, for the CO_2_ reduction route, the reduced form of the co‐factor, namely, NADH, is necessary. The group of Cazelles has investigated such reactions for the opposite redox pathway. In their work, they screen three different possibilities for regenerating NADH from NAD^+^, which is obtained from the dehydrogenase‐catalyzed reduction of CO_2_ to methanol. Their approaches comprise two techniques involving the use of phosphite dehydrogenase and glycerol dehydrogenase as enzymes and one photocatalytic approach with the use of a chloroplast‐containing photosystem.^60^

**Figure 3:**
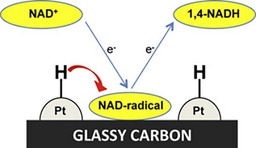
Electrochemical regeneration of NADH at a Pt‐modified glassy carbon electrode, as presented by Omanovic et al. Figure reproduced with permission from Ref. 62.

The group of Omanovic et al. has presented a study on converting NAD^+^ into NADH by using an electrochemical approach with a ruthenium‐modified glassy carbon electrode.^61^ Furthermore, they modify a glassy carbon electrode with Pt and Ni for the same purpose (Figure 3).^62^ Radical formation from NAD^+^, however, is critical, as this could give rise to the formation of dimers. Desired reduction reactions might not be performed quantitatively or might be hindered. Therefore, formation of dimers has to be prevented and either solution or electrode surface reactions may play a key role in preventing the coupling of radicals to afford dimers; this addresses the reaction towards the reduction of NAD^+^ to the active NADH isomer. Very recent results of this group show the utilization of bare and Ir–Ru oxide modified Ti electrodes for the electrochemical regeneration of NADH for over 80 % recovery.^63^, ^64^

Another electrochemical approach has been presented by Minteer and co‐workers. They use dehydrogenase enzymes coupled to an electrode for the reduction of CO_2_ and subsequent regeneration of NADH. They report improved efficiencies if another enzyme, carbonic anhydrase, is added.^65^

It is not only metals such as Ti, Ru, Ni, and Pt that have turned out to serve as suitable catalysts to regenerate NADH. Moreover, besides the electrochemical approach, photochemical techniques have also evolved. The groups of Dibenedetto and Aresta show the regeneration of NADH by using sodium dithionite for chemical conversion and zinc sulfide (ZnS) derivatives as a blank material or modified with Ru for photochemical applications.^54^ In other work, they show the photochemical regeneration of NADH by using a Rh–bipyridine catalyst. In both approaches, they investigate NADH regeneration coupled to CO_2_ reduction to methanol by using encapsulated enzymes.^66^

**Figure 4:**
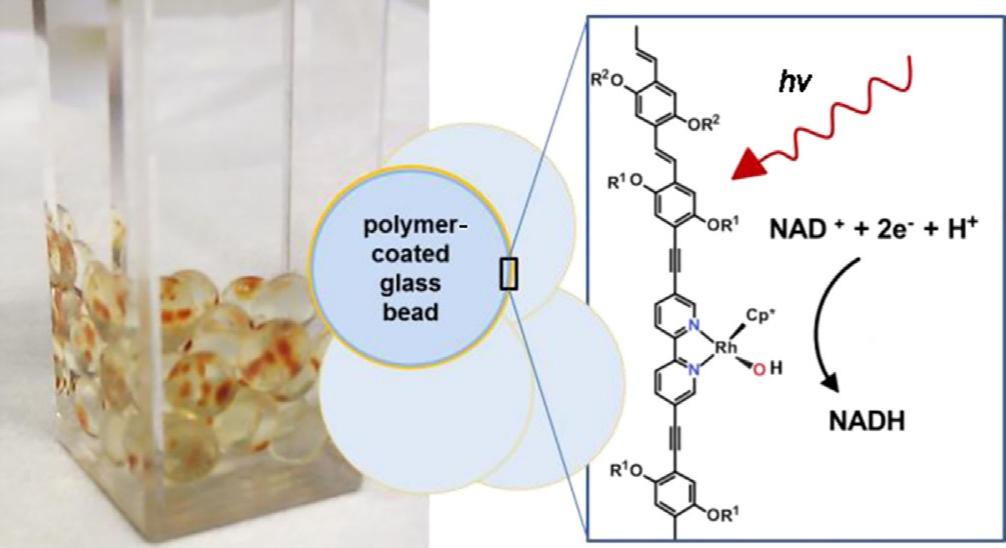
Glass beads coated with a Rh‐catalyst‐based polymer for the photochemical regeneration of NADH from NAD^+^, as displayed by Oppelt et al. Figure reproduced with permission from Ref. 68.

Also, Oppelt et al. focus on the utilization of Rh‐based complexes for the regeneration of NADH and NADH derivatives. They regenerate BNADH, a simplified NADH derivative, with the aid of a tin porphyrin complex, ethylenediaminetetraacetic acid (EDTA) or triethanolamin (TEOA), and a Rh complex. Moreover, they show the immobilization of a Rh‐complex‐based polymer on glass beads for the photoregeneration of NADH (Figure 4).^67^, ^68^

## 4 Enzymatic Electrocatalysis for CO_2_ Reduction

Co‐factor regeneration represents one strategy towards reducing the costs of enzymatic processes. Co‐factor recovery is indeed a feasible and promising approach to make enzyme‐catalyzed reactions less expensive and more efficient. However, an even more practical method would be the substitution of co‐factors as electron and proton suppliers. A different idea, gaining particular interest, is waiving the use of co‐enzymes and co‐factors. As such substances are responsible for the donation of electrons and protons in the case of reduction reactions, co‐factor substitution can only become affordable if techniques that take over the task of providing charges are found. For this purpose, photo‐ and electrochemical strategies have primarily been found to be suitable. Moreover, to develop this idea further, renewable energies could play an important role as energy sources for such methods. At best, the combination of biocatalyzed CO_2_ reduction to a fuel, driven by a renewable energy source, could provide a sustainable technique for energy storage.

In some work, photochemical investigations have been made by utilizing light sources to deliver charges.^30^ In 1984, Parkinson and Weaver presented results on photoelectrochemical approaches with the use of a p‐type potentiostatically driven InP photocathode in a two‐compartment cell without the requirement of any co‐factor. By using incident light, they report the photogeneration of methylviologen (MV^+^), which can serve as a reduction equivalent. CO_2_ is reduced to formic acid with FDH as the catalyst, whereas MV^+^ is oxidized but subsequently regenerated at the semiconductor electrode.^69^ Mandler and Willner describe the photoreduction of CO_2_ to formate by using visible light and Pd colloids as the catalyst.^70^ Also, Woolerton et al. report on the photoreduction of CO_2_. They use enzyme‐modified metal nanoparticles to catalyze the reduction of CO_2_. An additional photosensitizer is oxidized by transferring the electrons for the reduction, and it is then regenerated in an extra step.^71^, ^72^, ^73^ Baran et al. have recently published work on photocatalytic CO_2_ reduction by using a synthesized p‐type CuI semiconductor. They attribute the favorable photoreduction of CO_2_ to the fact that the conduction band edges are lower than those of n‐type semiconductors.^74^

However, whereas photoelectrochemical and photochemical methods require in situ incident light, electrochemical techniques would be flexible on the source of energy (e.g., sun or wind).

**Scheme 2:**
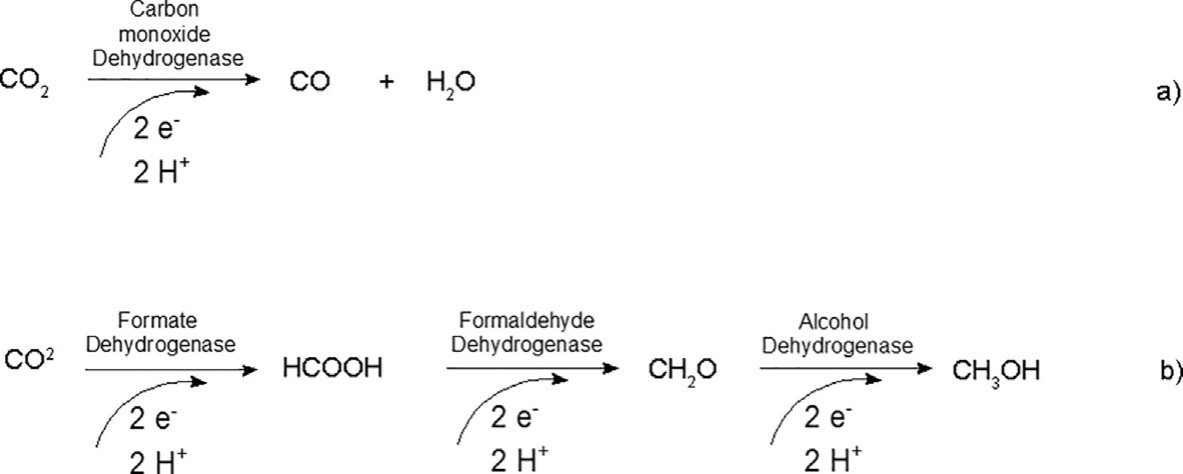
Reaction steps for direct electron injection in the electroenzymatic reduction of a) CO_2_ to CO with carbon monoxide dehydrogenase and b) CO_2_ to CH_3_OH through a three‐step cascade of dehydrogenases without any co‐factors.

Electroenzymatic processes, without any sacrificial electron donors or mediators, entail direct electron injection into the enzymes. The challenge is to introduce charges to the active site of the enzyme in a manner similar to that in photochemical approaches. Therefore, the naturally occurring intermediate state with the co‐factor, which is required for charge transfer, would be mimicked. Scheme 2 demonstrates the possible reduction route with direct electron injection from an electrode instead of co‐factors as the electron and proton donors.

Consequently, in electroenzymatic processes electrons are provided directly from an electrode and from an external energy source and protons have to be delivered from aqueous electrolyte solutions.

In 1993, Pantano and Kuhr presented their work on dehydrogenase‐modified carbon‐fiber microelectrodes. They use these electrodes to electrochemically monitor the formation of NADH, which serves as an electron mediator.^75^

**Figure 5:**
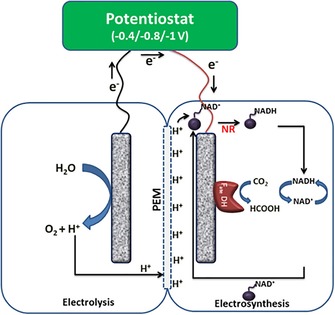
Reactor design shown by Srikanth et al. Combination of CO_2_ reduction and electrochemical NADH regeneration is presented. PEM=proton exchange membrane, NR=neutral red. Figure reproduced with permission from Ref. 77.

Similarly, Srikanth et al. present an approach for electrochemical CO_2_ reduction to formate catalyzed by formate dehydrogenase immobilized on an electrode. Besides CO_2_ reduction, NADH is regenerated in the electrochemical system at the same time (Figure 5).^76^

Also, the group of Yoneyama et al. have realized the potential in using enzymes as electrocatalysts. The focus of their work is on the electrochemical fixation and conversion of CO_2_. Especially, in their later work they show, as one of the first groups, the electrochemical addressing of dehydrogenase enzymes without the requirement of any co‐factor. They present the reduction of CO_2_ to methanol with methylviologen as an electron shuttle.^77^, ^78^

Methylviologen also serves as a supporting mediator for electron transfer in the work of Shin et al. They investigate the electrochemical application of carbon monoxide dehydrogenase. As a highly interesting result, the electroenzymatic reduction of CO_2_ to CO is performed at low overpotentials at 0.57 V versus the normal hydrogen electrode (NHE).^79^

Wang and co‐workers also focus on investigating carbon monoxide dehydrogenase. In their study, they screen two different carbon monoxide dehydrogenases by using protein‐film electrochemistry. Measurements are performed in the presence of CO_2_, and the influence of the different inhibitors is shown by comparing both dehydrogenases.^80^ Furthermore, calculations on the potential of such electroenzymatic approaches with carbon monoxide dehydrogenase and the role of the metals in the reduction of CO_2_ to CO have been published by Hansen et al.^81^

**Figure 6:**
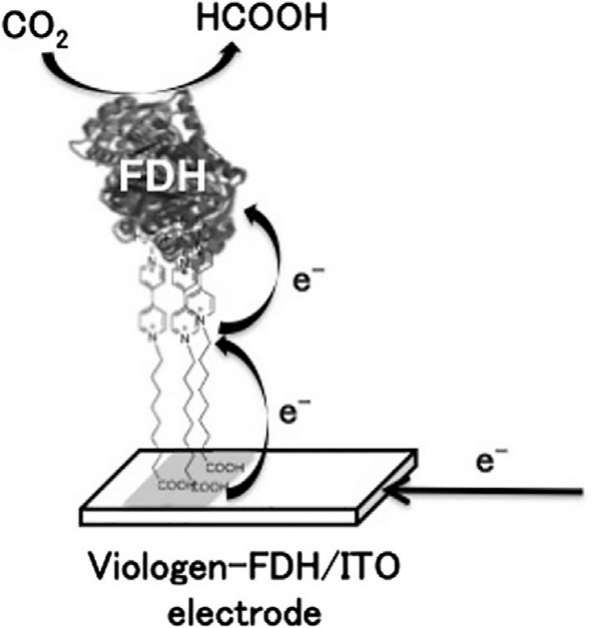
Covalent immobilization of FDH on an electrode by attaching it to methylviologen, which further serves as an electron shuttle, as reported by Amao et al. Figure reproduced with permission from Ref. 83.

Referring to methylviologen as an electron shuttle for electroenzymatic reactions, Amao and Shuto describe a similar approach. Differently, they couple formate dehydrogenase directly to methylviologen with a long alkyl chain, which is then linked to an indium tin oxide (ITO) electrode for an artificial photosynthesis approach, also comprising CO_2_ reduction to formate (Figure 6).^82^

Direct electron transfer without any shuttle has been studied by Razumiene et al. For their experiments, they use pyrroloquinoline quinone (PQQ)‐dependent alcohol dehydrogenase immobilized on carbon electrodes. An increase in the oxidative current is only observed if ethanol is added to the electrochemical system, which proves direct electron injection and electroenzymatic oxidation without any co‐factor or shuttle.^83^

Periasamy and co‐workers have also investigated ethanol oxidation by using alcohol dehydrogenase in an electrochemical system. In their approach, alcohol dehydrogenase is immobilized on glassy carbon, and the electrode is further coated with toluidine blue and Nafion to prevent leaching of the ADH.^84^

An electrochemical approach to address multienzyme cascades instead of single enzymes has been presented, for example, by the group of Minteer et al. They show direct electron transfer from enzymes for biofuel cells. Furthermore, they also show direct electron injection into dehydrogenase enzymes (i.e., FDH, F_ald_DH, and ADH) for methanol production coupled to NADH regeneration, as previously discussed.^65^, ^85^

**Figure 7:**
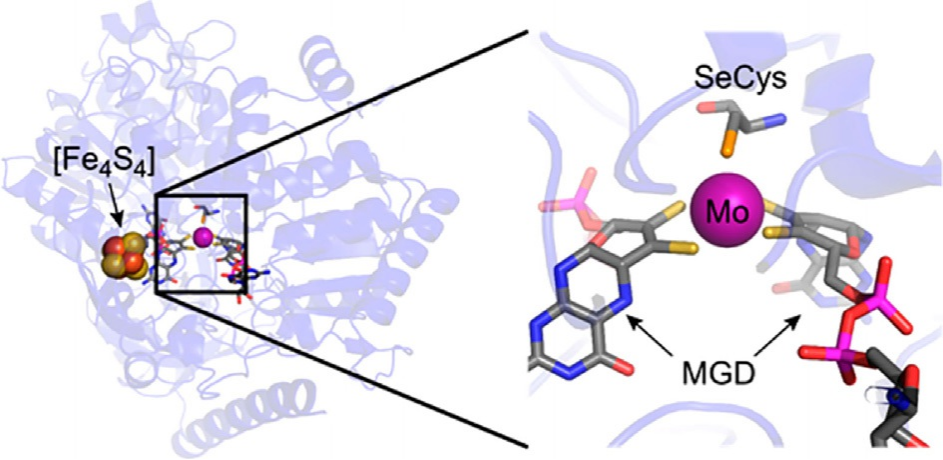
Proposed improved electron transfer resulting from the molybdenum active site of formate dehydrogenase for the reduction of CO_2_ to formate, as reported by Bassegoda et al. MGD=molybdopterin guanine dinucleotides. Figure reproduced with permission from Ref. 88.

Important work toward direct electron injection into enzymes for the heterogeneous, electroenzymatic reduction of CO_2_ without the requirement of any co‐factors or mediators was done by Reda et al. They demonstrate adsorption of tungsten‐containing formate dehydrogenase on glassy carbon. Using this enzyme electrode, they describe CO_2_ reduction to formate at reduction potentials below −0.8 V versus Ag/AgCl to yield faradaic efficiencies of 97 % and higher. Furthermore, they suggest an electron‐transfer mechanism among the electrode, the enzyme, and CO_2_ for the subsequent reduction reaction.^86^ Similarly, Bassegoda et al. use formate dehydrogenase with a molybdenum active site for the reversible conversion of formate and CO_2_. They suggest that the molybdenum‐containing FDH is even more electrochemically active than a tungsten‐containing FDH (Figure 7).^87^

**Figure 8:**
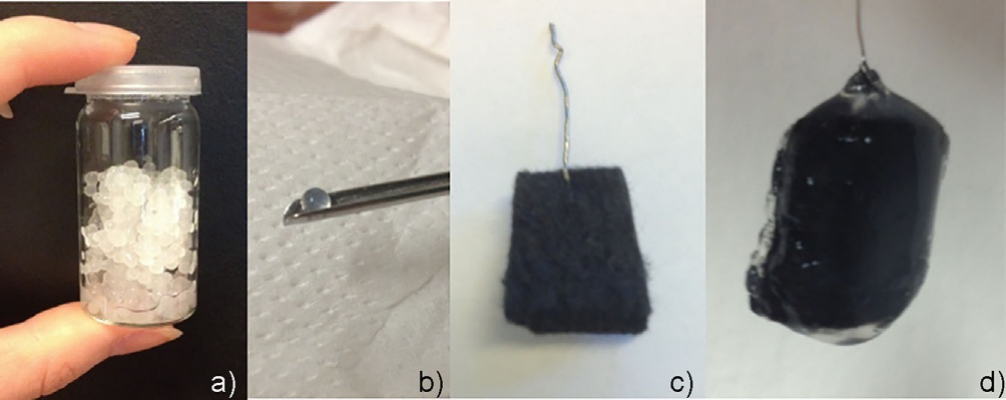
Preparation procedure for the immobilization of enzymes on a carbon felt electrode. a) Encapsulation of dehydrogenases in alginate–silicate hybrid gel. b) Resulting enzyme containing gel bead after precipitation. c) Blank carbon felt electrode that is soaked with the alginate–silicate mixture and precipitated in CaCl_2_. d) Alginate‐covered carbon felt electrode after precipitation.

Following the idea of catalytic electrodes for heterogeneous electrochemical CO_2_ reduction, Schlager et al. have recently described the immobilization of dehydrogenases encapsulated in an alginate‐based matrix on a carbon felt electrode (Figure 8).

**Figure 9:**
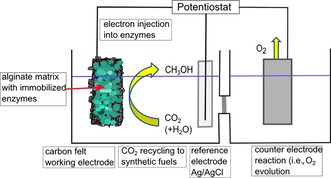
Scheme for the setup of an electrochemical cell for electroenzymatic CO_2_ reduction. Electrons are injected directly into the enzymes, which are encapsulated in alginate–silicate hybrid gel (green) and immobilized on a carbon felt working electrode. CO_2_ is reduced to methanol at the working electrode. Oxidation reactions take place at the counter electrode.^89^

Besides the immobilization of alcohol dehydrogenase alone for the conversion of an aldehyde into the corresponding alcohol, co‐immobilization of all three dehydrogenase enzymes for the reduction of CO_2_ to methanol has also been investigated (Figure 9). Both approaches deliver promising results with faradaic efficiencies of 10 % for the conversion of CO_2_ into methanol and even higher faradaic yields of up to 40 % for the single immobilized enzyme. Moreover, all experiments are performed without the addition of any co‐factor or electron mediator.^88^, ^89^

Specifically, approaches requiring no additional mediators or electron shuttles are of high interest. Reduced costs owing to such simplified processes combined with heterogeneous electrocatalysis to enable electrode reusability make such processes attractive for large‐scale applications. Studies on optimizing electroenzymatic processes for application in renewable energy storage and CO_2_ reduction are enormously evolving and pave the way towards sustainable fuel generation. Moreover, the feasibility of immobilizing enzymes on electrodes and subsequent direct electron injection offers the possibility for applications other than fuel generation, such as in the food and pharmaceutical industries.

## 5 Summary and Outlook

In this work, an approach towards CO_2_ utilization was presented. CO_2_ represents a carbon source and carbon feedstock that can be reduced to valuable chemicals and fuels. In the last decades, much work has been presented that discusses the feasibility of recycling CO_2_ and therefore substituting fossil carbon sources such as oil, gas, coal, and biomass. In particular, approaches mimicking natural processes have drawn attention, and techniques for the photocatalytic and electrocatalytic conversion of CO_2_ have been developed. However, most synthetic catalysts such as organic, metallic, and organometallic compounds that have been used to enable the reduction of CO_2_ to energy‐rich chemicals often do not yield high efficiencies or provide high selectivity to the desired products.

Therefore, the use of not only bioinspired but even biobased catalysts such as enzymes has especially gained interest in science. Biocatalysts and enzymes are known from natural CO_2_ reduction processes, for which they yield high efficiencies and selectivity under mild reaction conditions (e.g., ambient temperature and pressure, aqueous media at neutral pH). Nevertheless, such processes require electron and proton donors or co‐factors. In nature, such substances are regenerated in coupled reactions and, therefore, closed cycles. For the purpose of CO_2_ reduction in laboratory or industrial approaches, however, such co‐factors and equivalents are sacrificial and are, therefore, not practical because of their high costs.

Herein, we have given an overview of studies in which enzymes were used as catalysts for CO_2_ reduction. We showed different strategies focusing on co‐factor regeneration on the one hand and substitution of such co‐factors on the other hand. Different catalysts to recover the co‐factors, such as nicotinamide adenine dinucleotide, as well as photochemical and electrochemical approaches for direct electron injection into enzymes without the requirement of any co‐factors were shown. Such strategies make enzymes appealing for the purpose of CO_2_ conversion, as processes can be eased and costs can be reduced remarkably. We displayed recent results in this increasingly evolving field, representing approaches with high potential toward CO_2_ utilization and renewable energy storage at the same time and making enzymatic processes attractive for large‐scale applications.

## Biographical Information

Stefanie Schlager joined the group of Linz Institute of Organic Solar Cells (LIOS) in 2010 and finished her studies in technical chemistry and chemical engineering at the Johannes Kepler University in Linz in 2011. The topic of her first diploma thesis was on organic Schottky diodes and was followed by her second diploma thesis and PhD at LIOS, where she focused on the application of semiconductor electrolyte interfaces for application in electrochemical CO_2_ reduction as well as the biocatalytic and bioelectrocatalytic reduction of CO_2_. Since the beginning of 2016, she has been a postdoctoral assistant at LIOS with her scientific work based on the further development of microbial electrosynthesis and electroenzymatic conversion processes.

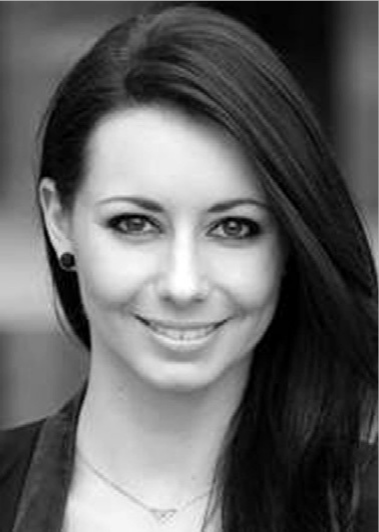

## Biographical Information

Prof. N. S. Sariciftci studied at the University of Vienna, Austria, and earned his PhD degree in physics in 1989. After a two‐year postdoctoral stay at the University of Stuttgart, Germany, he joined the Institute for Polymers and Organic Solids at the University of California, Santa Barbara, CA, working in the group of Prof. Alan J. Heeger. Since 1996 he has been the Ordinarius (chair) Professor for Physical Chemistry and the founding director of LIOS at the Johannes Kepler University in Linz. His major contributions are in the fields of photoinduced optical, magnetic resonance, and transport phenomena in semiconducting and metallic polymers and in organic and bioorganic semiconductors. In recent years, research on CO_2_ utilization has attracted his increasing interest. Sariciftci is a member of the Academy of Sciences in Austria (ÖAW) and has been awarded honorary doctorates and has received several prizes, among them the prestigious Wittgenstein Prize of Austria in 2012.